# An improved NFC device authentication protocol

**DOI:** 10.1371/journal.pone.0256367

**Published:** 2021-08-16

**Authors:** He-Jun Lu, Dui Liu

**Affiliations:** 1 The School of Big Data and Artificial Intelligence, Anhui Xinhua University, Hefei, Anhui, China; 2 Security Research Institute, Hangzhou Anheng Information Technology Co., Ltd., Hangzhou, Zhejiang, China; Civil Aviation University of China, CHINA

## Abstract

Aimed at the security authentication problem between Near Field Communication (NFC) devices, this paper uses the technology of asymmetric encryption algorithm, symmetric encryption algorithm, hash function, timestamp and survival period to improve the confidentiality, performance and security of the protocol. The symmetric encryption algorithm encrypts the transmission content, while the asymmetric encryption algorithm encrypts the shared key. The whole authentication process is secure, and the key distribution is secure. The improved NFC device authentication protocol can effectively resist the brute force attack, man-in-the-middle attack and replay attack in the authentication process, it can reduce the number of message transmission in the authentication process, improve the transmission efficiency, enhance the confidentiality, integrity, non-repudiation and improve the security of NFC device authentication.

## Introduction

In recent years, with the wide application of smart phones, people’s life and consumption patterns have been fundamentally changed, especially in the aspect of mobile payment, which is expected to replace credit card payment and cash payments. The way of mobile payment consumption has gradually become popular and is well known and accepted by the public [1–3]. NFC technology [4] is a very common way in the process of mobile payment. This technology uses the frequency of 13.56 MHz to work within a range of 10 cm. By using the ISO/IEC 18092 standard, NFC devices can work like ordinary contactless smart cards, and is now widely used in various fields [5–8]. Because NFC contains ISO/IEC 14443 standard, relay attack is feasible [9]. At the same time, more attention is paid to its transmission efficiency in the process of NFC communication, but the security issues in the process of communication are ignored, and faced with the risk on the penetration [10], especially the defects in authentication. This paper proposes an improved, efficient and secure NFC device authentication protocol.

Symmetric encryption algorithm [11,12], asymmetric encryption algorithm [13,14], hash function [15,16] and other related technologies are used in the protocol. In the whole protocol design process, man-in-the-middle attack [17], replay attack [18], brute force attack [19], data integrity and confidentiality [20,21] and other factors are comprehensively considered. Based on Lee et al. ’s research on NFC man-in-the-middle attack [22], Ceipidor et al.’ s research on NFC payment security [23], Thammarat et al. ’s research on NFC security lightweight protocol [24], Tung et al.’ s research on efficient NFC authentication scheme [25] and other relevant studies [26–31], an improved efficient and secure NFC device authentication scheme is proposed.

## Methodology

In this paper, the protocol uses symmetric algorithm to guarantee the security of NFC device Identity (ID) and random number in the transmission process, uses public key algorithm to realize shared key distribution and message authentication, and uses hash function to verify the integrity of messages, which is divided into two stages: registration stage and authentication stage. In the registration phase, a random number is generated by the NFC device, and a random number is generated by the Authentication Server (AS). By using the two random numbers, the two-factor authentication is realized and the brute force attack is prevented. At the same time, the survival period of the NFC device issued by the authentication server and the timestamp of the authentication stage are used to prevent the replay attack. The identifiers and explanations used in this protocol are as shown in Table 1.

**Table 1:** Identifiers and explanations used in the protocol.

Identifier	Interpretative Statement
**N**_**i**_	N is NFC device, i is NFC device number
**AS**	Authentication Server
**E**	Asymmetric Encryption Algorithm
**S**	Symmetric Encryption Algorithm (Shared key encryption algorithm)
**puk**	Public key of AS
**prk**	Private key of AS
**K**_**i**_	The Shared key between N_i_ and AS
**H**	Hash Encryption Algorithm
**Rn**_**i**_	The random number generated by N_i_
**Rn**_**Ai**_	The random number generated by AS
**SP**_**i**_	The survival period of N_i_
**TS**	Timestamps

### Registration phase

#### Step1 N_1_—> AS: RQE1, RQS1

N_1_ Sends the requests of RQE1 and RQS1 to AS.


$$RQE1=E_{puk}\left\{K_{1}\right\}$$ (1)

$$RQS1=SK_{1}\left\{IDN1,Rn_{1}\right\}$$ (2)

R represents the Registered stage. Q represents the Request message. E represents the use of asymmetric encryption algorithm. S represents the use of Shared Key encryption (symmetric encryption algorithm), and the number one represents the first message in the information of RQE1 and RQS1. N_1_ encrypts the shared key K_1_ through the public key of AS, and encrypts its own information IDN1 and random number Rn_1_ through the shared key and sends it to AS to complete the shared key distribution and registration request.

#### Step2 AS—> N1: RPE1, RPS1

After AS receives the message from N_1_, it decrypts RQE1 through its own private key to get K_1_, and then RQS1 is decrypted by using K_1_ to get Rn_1_ and IDN1. After registering the IDN1 and Rn_1_ of N_1_ in its database, AS generates Rn_A1_ and SP_1_, and sends RPE1 and RPS1 to N_1_.


$$RPE1=E_{prk}\left\{H\left(IDN1,Rn_{1}\right)\right\}$$ (3)

$$RPS1=SK_{1}\left\{SP_{1},H\left(Rn_{A1}\right)\right\}$$ (4)

P represents the response request message in the information of RPE1 and RPS1. Rn_A1_ is the random number generated by AS. SP_1_ is the survival period of N_1_ device. N_1_ needs to be authenticated within the SP_1_, and otherwise authentication fails.

**Fig 1:**
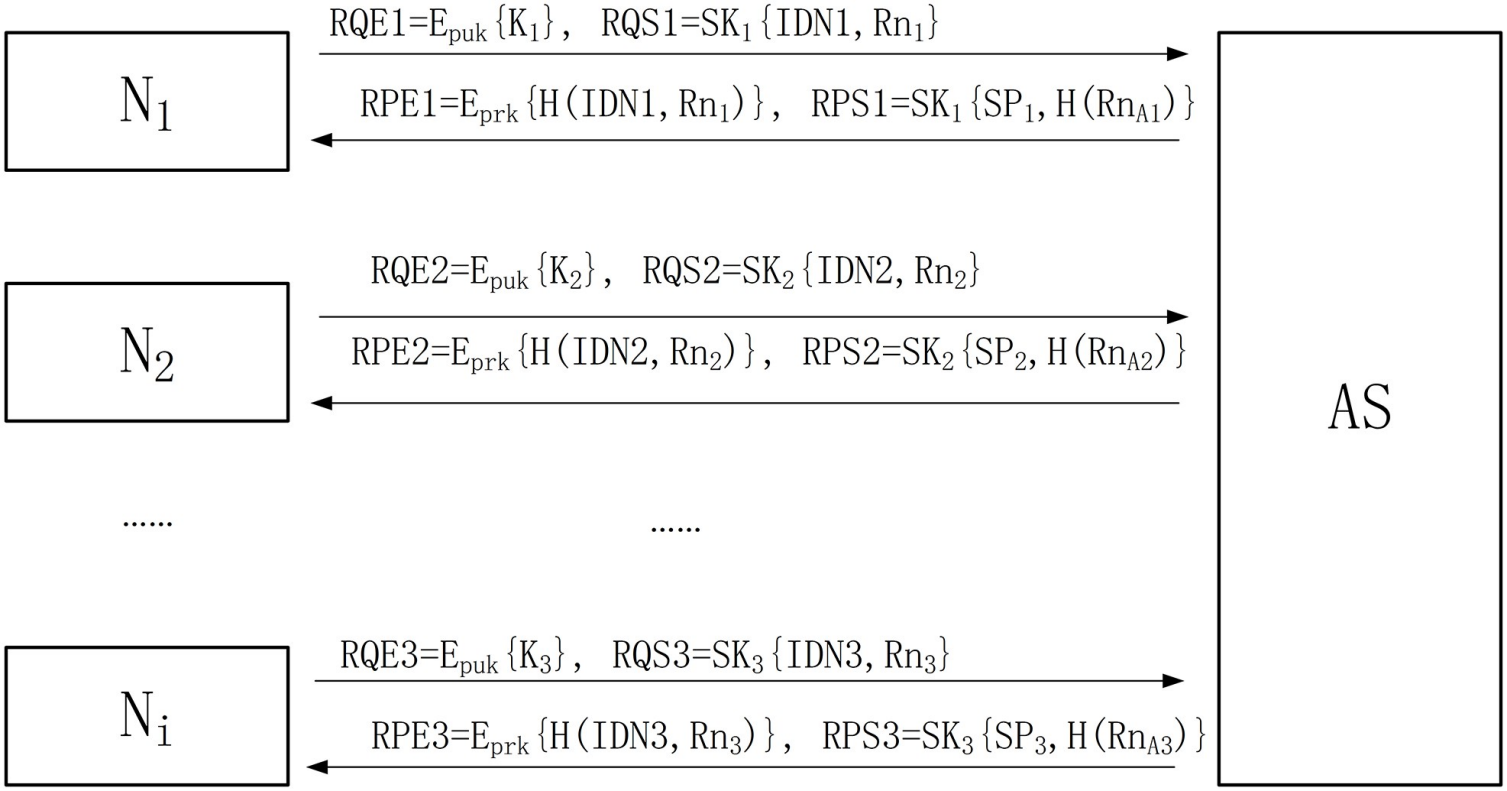
The working process of the registration phase.

When N_1_ receives RPE1 and RPS1, it decrypts RPE1 by using the public key of AS to obtain H(IDN1, Rn_1_), and then compares it with the H(IDN1, Rn_1_) generated by itself. If it is consistent, the message is indeed sent by AS and has not been changed in the transmission process. The verification is successful, then the registration stage is completed, otherwise the registration fails. The working process of the registration phase is shown in Fig 1.

### Authentication phase

#### Step1 N1—> N2: AQS1, AQH1

N_1_ sends AQS1 and AQH1 requests to N_2_, where "A" represents the authentication phase in the message of AQS1 and AQH1.


$$AQS1=SK_{1}\left\{IDN1,Rn_{1},H\left(Rn_{A1}\right)\right\}$$ (5)

$$AQH1=H\left\{IDN1,Rn_{1},H\left(Rn_{A1}\right)\right\}$$ (6)

N_1_ encrypts IDN1, Rn_1_ and H(Rn_A1_) using the shared key K_1_ to generate AQS1, and at the same time carries out the hash calculation to generate the hash value AQH1 to ensure the confidentiality and integrity of the information.

#### Step2 N2—> AS: AQS2, AQH2

After receiving the messages send by N_1_, N_2_ encrypts IDN2, Rn_2_, H(Rn_A2_) and AQS1 with its shared key with AS to generate AQS2. At the same time, the information in AQS2 and the value of H{IDN1, Rn_1_, H(Rn_A1_)} is used to generate the hash value AQH2 to ensure the data integrity in the transmission process. After that, the generated AQS2 and AQH2 are sent to AS.


$$AQS2=SK_{2}\left\{IDN2,Rn_{2},H\left(Rn_{A2}\right),SK_{1}\left\{IDN1,Rn_{1},H\left(Rn_{A1}\right)\right\}\right\}$$ (7)

$$AQH2=H\left\{IDN2,Rn_{2},H\left(Rn_{A2}\right),H\left\{IDN1,Rn_{1},H\left(Rn_{A1}\right)\right\}\right\}$$ (8)

#### Step3 AS—> N2: APS1, APE1, APE2

After receiving the request from N_2_, the AS decrypts AQS2 information through K_2_ and K_1_, and obtains the information of IDN2, Rn_2_, H(Rn_A2_), IDN1, Rn_1_ and H(Rn_A1_). If this information does not match the data in the database, the authentication process is terminated. If it is consistent, the information in AS database is used to generate H{IDN2, Rn_2_, H(Rn_A2_), H(IDN1, Rn_1_, H(Rn_A1_)}}. If it is consistent with the hash value sent by N_2_, the authentication will continue, otherwise, the authentication will be terminated.

After AS verifies N_1_ and N_2_, it sends responses APS1, APE1, APE2 to N_2_. Note that the information in APS1, APE1, and APE2 all use the information in the database of AS. TS_1_ is the timestamp.


$$APS1=SK_{2}\left\{H\left\{IDN2,Rn_{2},H\left(Rn_{A2}\right)\right\},TS_{1},SK_{1}\left\{H\left\{IDN1,Rn_{1},H\left(Rn_{A1}\right)\right\}\right\}\right\}$$ (9)

$$APE1=E_{prk}\left\{H\left(IDN1\right)\right\}$$ (10)

$$APE2=E_{prk}\left\{H\left(IDN2\right)\right\}$$ (11)

#### Step4 N2—> N1: APS2, APE1

After receiving the response from AS, N_2_ decrypts APE2 through the public key issued by AS to verify whether the H(IDN2) is the same as its own ID hash value to confirm whether the message comes from AS. If the verification fails, the authentication is terminated. If it succeeds, SK_2_ is used to decrypt APS1 to obtain the messages of H{IDN2, Rn_2_, H(Rn_A2_)} and TS_1_. N_2_ uses TS_1_ to verify the validity of the message. When the verification of TS_1_ passed, the H{IDN_2_, Rn_2_, H(Rn_A2_)} is calculated to be consistent with those received. If TS_1_ verification fails, the authentication will be terminated. At the same time, if the verification of H{IDN2, Rn_2_, H(Rn_A2_)} fails, the authentication will also be terminated.

When N_2_ confirms that the received message is correct, APS2 and APE1 are sent to N_1_.


$$APS2=SK_{1}\left\{H\left\{IDN1,Rn_{1},H\left(Rn_{A1}\right)\right\}\right\}$$ (12)

$$APE1=E_{prk}\left\{H\left(IDN1\right)\right\}$$ (13)

After N_1_ receives the response from N_2_, it first obtains H(IDN1) by calculating APE1 with public key, and then compares the obtained hash value with itself hash value to verify their consistency. If they are consistent, the source and integrity authentication are completed.

**Fig 2:**
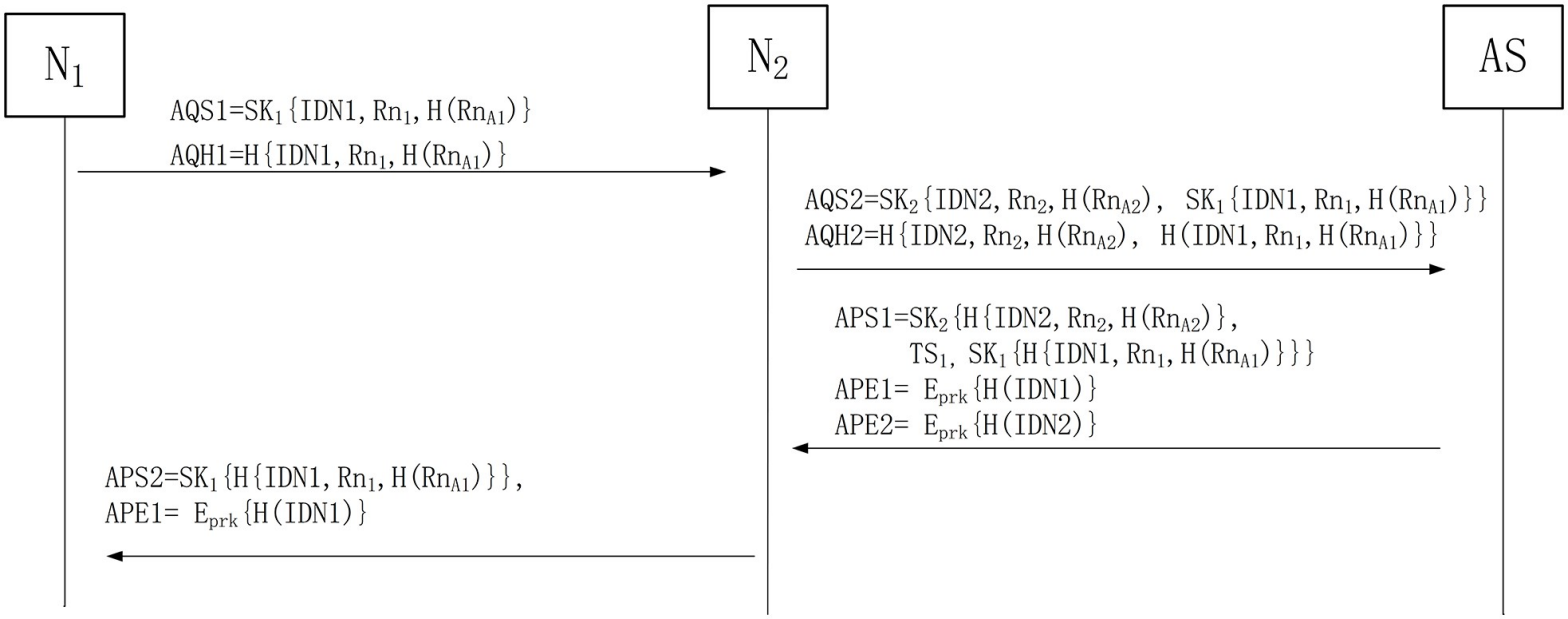
The working process in the authentication phase.

N_1_ uses SK_1_ to solve APS2 to obtain H{IDN1, Rn_1_, H(Rn_A1_)} and then compared with the H{IDN1, Rn_1_, H(Rn_A1_)} generated by itself using local data for matching verification. If they are consistent, the authentication is completed. Otherwise, the authentication fails and the authentication is terminated. The working process of certification phase is shown in Fig 2.

## Results

### Prevent man-in-the-middle attacks

In this paper, ciphertext transmission is adopted. In the whole process of the protocol, including two phases of registration and authentication, the middleman cannot obtain the effective plaintext information. Suppose the middleman is located in N_1_And N_2_, APE1 cannot be generated, because he does not know the key of AS and the value of IDN1, so it will not pass the fourth step in the authentication phase. If the middleman is located at N_2_ and AS, because he doesn’t know the shared key K_2_ and the private key of AS, the middleman will not generate APS1, APE1 and APE2, and TS will also be able to defend against man-in-the-middle attacks between N_2_ and AS to some extent.

### Prevent replay attacks

In this paper, random numbers Rn_i_ and Rn_Ai_, message lifetime, timestamp and other technologies are used. Compared with the scheme proposed by Lee et al., Ceipidor et al., Thammarat et al., it can effectively achieve the purpose of preventing replay attacks.

### Prevent brute force attacks

In this paper, the random numbers Rn_i_ generated by the NFC device and the random numbers Rn_Ai_ generated by the AS are used. At the same time, symmetric encryption, asymmetric encryption and hash encryption technologies are adopted, making brute force cracking extremely difficult. Compared with the scheme proposed by Lee et al., Tung et al., the scheme is more secure and reliable on the whole process.

### Ensure data integrity

In this paper, there are corresponding hash values in each step of the registration phase and authentication phase, which can fully guarantee the integrity of the data. In the second step of authentication stage, dual hashing integrity authentication is used, which can ensure the integrity of data better than other schemes.

### Ensure data confidentiality

In this paper, asymmetric algorithm is used to encrypt the key of symmetric algorithm, and symmetric algorithm is used to encrypt the data, which not only ensures the confidentiality, but also ensures the efficiency of the whole protocol. The whole process is encrypted, so that all the data obtained by the attacker are ciphertext. Compared with the scheme proposed by Ceipidor et al. and Tung et al., the data confidentiality is stronger and more secure.

### Mutual authentication

In the second step of the authentication phase, AS verifies the consistency of the ID and Rn_1_, Rn_A1_, Rn_2_ and Rn_A2_ by receiving requests from N_1_ and N_2_, and then uses the data in the local database to conduct hash calculation in response to N_1_ and N_2_. N_1_ and N_2_ use their own Rn_1_, Rn_A1_, Rn_2_ and Rn_A2_ to conduct hash calculation and verify whether they are consistent and achieve the purpose of mutual authentication.

## Discussion

### Confidentiality and performance analysis

Compared with other schemes, this paper comprehensively uses symmetric encryption algorithm, asymmetric encryption algorithm and hash algorithm to achieve high confidentiality. In terms of message transfer operation frequency, the protocol in this paper completes the entire authentication process with the minimum message transmission times, which is efficient and safe. Comparison of confidentiality and performance analysis are shown in Table 2.

**Table 2:** Comparison of confidentiality and performance analysis.

Operating	Our scheme	Lee et al.	Ceipidor et al.	Thammarat et al.	Tung et al.
**Symmetric Encryption algorithm**	8	4	-	3	-
**Asymmetric Encryption algorithm**	6	1	2	-	-
**Hash algorithm**	8	3	3	9	9
**Message transmissions**	6	8	7	8	7

### Safety analysis

This paper compares with other schemes in security aspects such as confidentiality, prevention of man-in-the-middle attack, prevention of replay attack, prevention of brute force attack, integrity, mutual authentication, etc., as shown in Table 3. It can be seen that the protocol in this paper can complete the mutual authentication of NFC devices and ensure the security at the same time. The message transmitted in the registration and authentication stages is encrypted throughout, which is more secure than the protocol with others.

**Table 3:** Comparison of safety analysis.

Security	Our study	Lee et al.	Ceipidor et al.	Thammarat et al.	Tung et al.
**Confidentiality**	Yes	No	No	No	No
**Preventing man-in-the-middle attacks**	Yes	-	-	Yes	Yes
**Prevent replay attacks**	Yes	No	No	Yes	Yes
**Prevent brute force attacks**	Yes	-	-	Yes	Yes
**Integrity**	Yes	No	-	Yes	Yes
**Mutual authentication**	Yes	-	Yes	Yes	Yes

Note that the "-" in the table indicates that this study claims that the solution can provide this security attribute, but other studies believe that the solution still lacks this security attribute.

## Conclusion

A secure and efficient authentication scheme between NFC devices is proposed in this paper. The whole ciphertext transmission can not only be used for communication between mobile NFC devices, but also for secure communication between NFC devices and smart cards. At the same time, the scheme uses the timestamp, survival period and other technologies to solved the man-in-the-middle attack, replay attack and other problems. The hash algorithm is used to ensure the data integrity in the transmission process. The asymmetric encryption algorithm is used to solve the problem of message source authentication and shared key distribution. The symmetric encryption is used to make the protocol more efficient. In this protocol, the number of interactive information transmission between devices is reduced as much as possible, and the messages transmitted in both the registration stage and the authentication stage are all encrypted, which makes the whole system more secure.
